# Bioactivity-guided isolation of antioxidant and anti-hepatocarcinoma constituents from *Veronica ciliata*

**DOI:** 10.1186/s13065-016-0172-1

**Published:** 2016-05-03

**Authors:** Li Yin, Qiuxia Lu, Shancai Tan, Lisheng Ding, Yiran Guo, Fang Chen, Lin Tang

**Affiliations:** College of Life Sciences, Sichuan University, Key Laboratory of Bio-resources and Eco-environment, Ministry of Education, No.24 South Sect. 1, Yihuan Road, Chengdu, People’s Republic of China; National and Local Joint Engineering Laboratory for Energy Plant Bio-oil Production and Application, Chengdu, Sichuan China; Key Laboratory of Mountain Ecological Restoration and Bioresource Utilization, Chengdu Institute of Biology, Chinese Academy of Sciences, Chengdu, People’s Republic of China

**Keywords:** Scrophulariaceae, *Veronica ciliata*, Antioxidant, Anti-hepatocarcinoma, Iridoid glycosides, Bioactivity-guided screening

## Abstract

**Background:**

*Veronica ciliata* Fisch., widely distributed in western China, has been traditionally used in Tibetan Medicine as a treatment for hepatitis, cholecystitis, rheumatism, and urticaria. However, *V. ciliata* Fisch. has not been subjected to detailed chemical constitution analysis and the bioactive studies were restricted to its crude extracts. It is necessary to investigate the active chemical components of these extracts and identify their biological effects.

**Results:**

Four iridoid glycosides, (veronicoside, cataposide, amphicoside, and verminoside) were isolated from the ethyl acetate fraction. Among these compounds, veronicoside and verminoside were isolated for the first time from this plant. These compounds exhibited strong antioxidant activity and inhibitory activity on HepG2 cell proliferation. The antioxidant activity of verminoside was equal to Vc. Cataposide, amphicoside and verminoside had stronger anti-hepatocarcinoma activity than 5-fluorouracil.

**Conclusions:**

Four iridoid glycosides,(veronicoside, cataposide, amphicoside and verminoside) were isolated from the extract of *V. ciliata* Fisch. using bioassay-guided screening.Among these compounds, veronicoside and verminoside were isolated for the first time from this plant. The above results indicated that these compounds were the active chemical components responsible for the antioxidant and anti-hepatocarcinoma properties of *V. ciliata* Fisch. The underlying mechanism of their bioactivity is worthy of further investigation.

**Graphical abstract:**
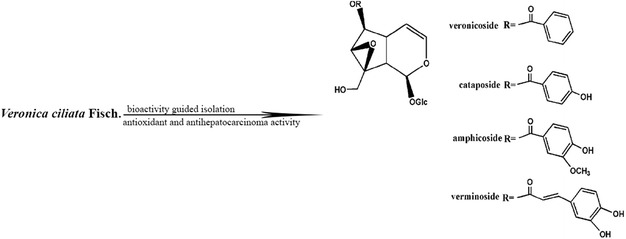
Bioactivity-guided isolation of antioxidant and anti-hepatocarcinoma constituents from *Veronica ciliata*

## Background

Liver cancer is common in sub-Saharan Africa and Southeast Asia and is currently the most common type of cancer in many countries in these regions [1]. A large number of medicinal plants have been tested and found to contain active compounds with curative proper properties against liver cancer [2–4]. *Rehmannia glutinosa* and *Scrophularia ningpoensis* Hemsl. (Scrophulariaceae) were used for the treatment of liver diseases and have a long history [5, 6]. Picroliv is a standardized fraction of alcoholic extract from *Picrorhiza kurroa* (Scrophulariaceae) and significantly protects against hepatic damage [7]. Therefore, scrophulariaceous plants are worth studying to explore their anti-hepatocarcinoma activities.

*Veronica ciliata* Fisch., belonging to Scrophulariaceae, is a psychrophyte from the northwest territories, northern Sichuan, and the Tibetan autonomous region (China). In China, this plant has been traditionally used in Traditional Chinese Medicine to treat hepatitis, cholecystitis, rheumatism and urticarial [8]. The extracts of *V. ciliata* Fisch. were reported to have strong antioxidant activities and significantly protective effects against acute hepatotoxicity induced by CCl_4_ [9]. Five iridoid glycosides and three derivatives of benzoic acid have been isolated from *V. ciliata* Fisch. [10]. However, *V. ciliata* Fisch. has not been subjected to detailed chemical constitution analysis and the bioactive studies were restricted to its crude extracts. It is necessary to investigate the active chemical components of these extracts and identify their biological effects. Given that there are no reports of the anti-hepatocarcinoma activity of *V. ciliata* Fisch., this study examined the antioxidant activity and anti-hepatocarcinoma activity of crude extracts and four fractions of *V. ciliata* Fisch. on hepatoma cell HepG2 proliferation. Subsequently, four iridoid glycosides with these bioactivities, especially the anti-hepatocarcinoma activity on HepG2 cells, were identified from *V. ciliata* Fisch. Among these compounds, veronicoside and verminoside were isolated for the first time from this plant.

## Results and discussion

### Structure identification of the purified compounds

**Fig. 1:**
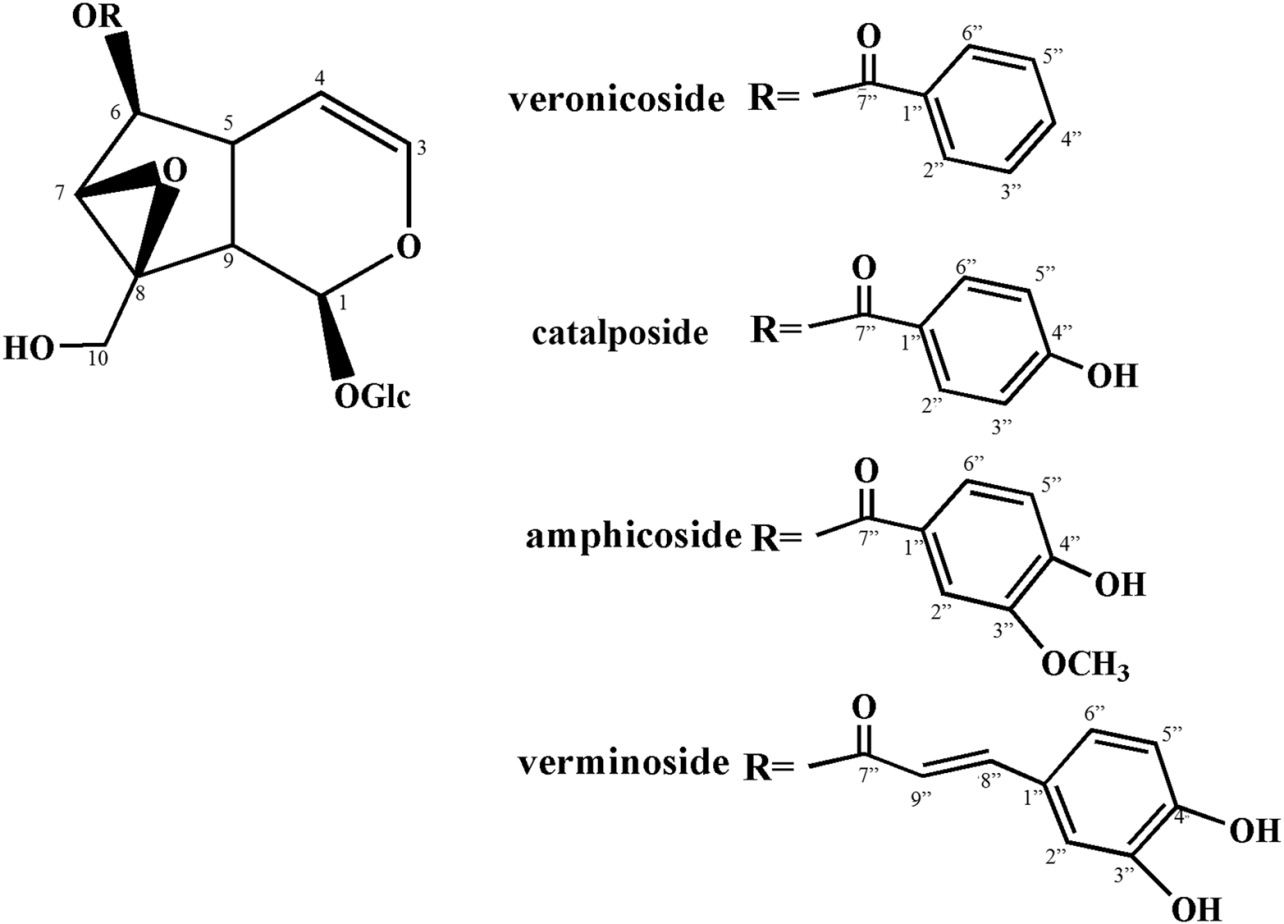
Compounds and their chemical structures isolated from *V. ciliata* Fisch.

To investigate the chemical constituents of *V. ciliata* Fisch., four compounds were obtained after isolation and purification. On the basis of spectroscopic analysis, and comparison with the previously reported spectral data [10–14], the structures of these compounds were identified as veronicoside, cataposide, amphicoside, and verminoside. Among all of these compounds, veronicoside and verminoside were isolated from *V. ciliata* Fisch. for the first time. The purity of these compounds were above 95 %. The chemical structures of these compounds were shown in Fig. 1^13^C-NMR of four compounds were shown in Table 1.

Compounds 1–4 showed similar NMR spectral patterns except for the phenylpropanoid or benzoic acid derivatives. Fifteen signals for catalpol were detected in the ^13^C-NMR spectra of compounds 1–4, with similar chemical shift values (Table 1). The ^1^H- and ^13^C-NMR spectra of compounds 1–3 revealed signals for benzoyl, p-hydroxybenzoyl, and vanilloyl, respectively. The structures of these compounds were identified as veronicoside, cataposide, and amphicoside, on the basis of a comparison with the previously reported spectral data [11, 15]. The phenylpropanoid moiety for compound 4 was identified from its spectral data which showed caffeoyl groups. Thus, the structure of compound 4 was identified as verminoside by comparison with their previously reported spectral data [16]. The spectral data of amphicoside and catalposide were compared with the previously reported data [10].

**Table 1:** ^13^C-NMR data of compouds 1–4

Carbon	1	2	3	4
1	92.8	92.9	94.4	94.4
3	141.3	141.2	142.4	142.4
4	101.4	101.6	102.5	102.6
5	35.4	35.3	36.4	36.5
6	80.2	80.1	80.9	80.9
7	58.1	58.3	59.4	59.4
8	65.8	65.7	66.7	66.7
9	41.8	41.7	42.8	42.8
10	58.4	58.5	59.8	59.8
1′	97.9	97.8	98.9	99.0
2′	73.4	73.3	74.8	74.9
3′	77.4	77.5	78.9	78.9
4′	70.3	70.4	72.1	72.0
5′	76.4	76.5	77.9	77.9
6′	61.4	61.5	62.8	62.9
1″	133.6	119.4	121.5	126.8
2″	129.3	131.6	113.2	115.2
3″	128.8	115.4	149.2	147.1
4″	129.3	162.4	153.6	150.2
5″	128.6	115.4	116.4	114.8
6″	129.9	131.6	125.4	116.6
7″	165.8	165.5	168.1	123.4
8″				147.7
9″				169.2
OCH_3_			56.8	

### In vitro antioxidant activity assays

**Fig. 2:**
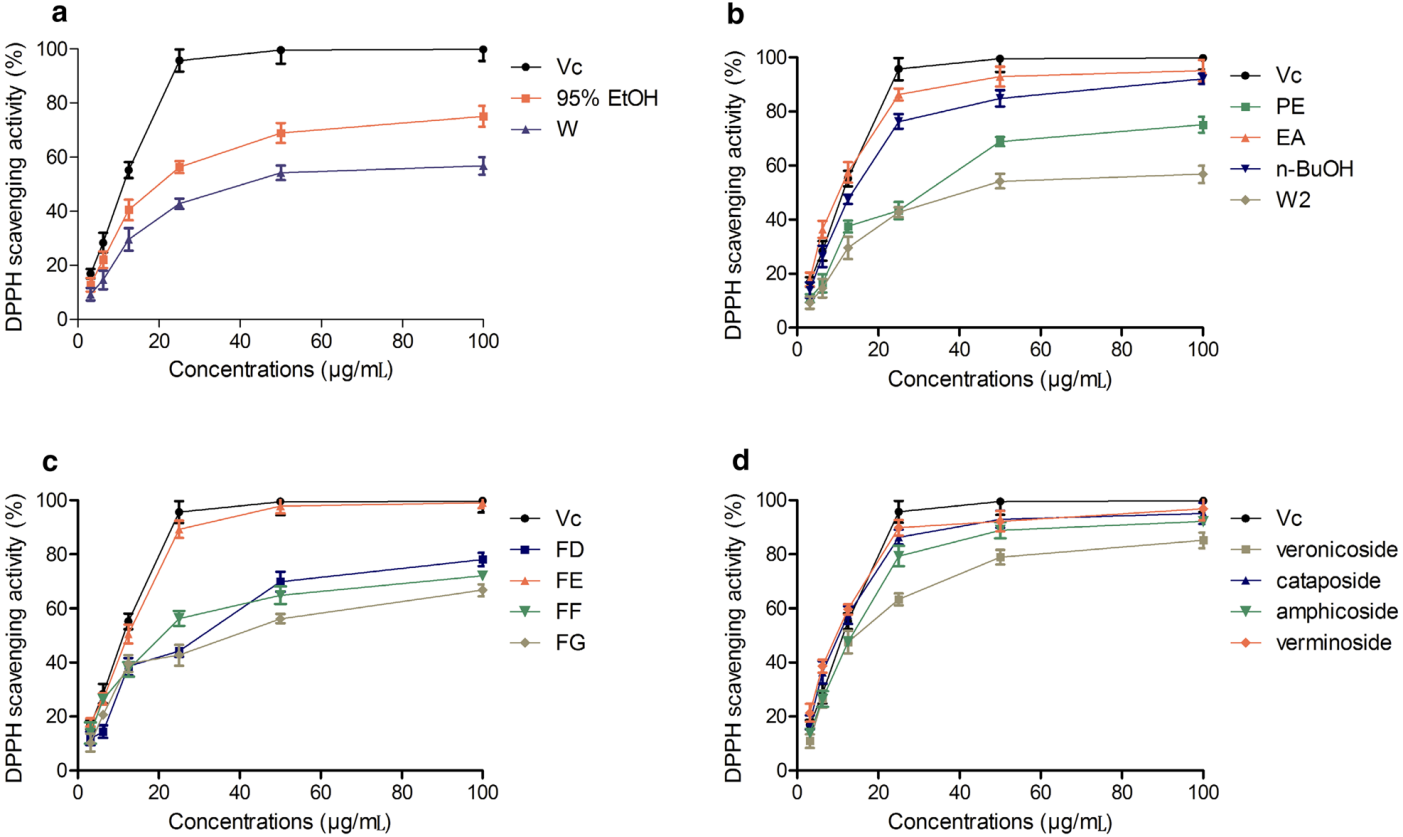
DPPH free radical scavenging ability of the (**a**) 95 % ethanol extract (95 % EtOH), water extract (W); **b** petroleum ether fraction (PE), ethyl acetate fraction (EA), *n*-butanol (*n*-BuOH) fraction, and water fraction (W2) from 95 % ethanol extract; **c** fractions (FD, FE, FF, FG) from EtOAc fraction; and **d** veronicoside, cataposide, amphicoside and verminoside. Results are mean ± SD

The DPPH free radical scavenging ability of each sample is shown in Fig. 2. All of the extracts exhibited DPPH radical scavenging activity, and the scavenging action of the 95 % ethanol extract was higher than that of the water extract (Fig. 2a). The EtOAc fraction showed more significant scavenging activity than the petroleum ether, *n*-BuOH, and water fractions and it was comparable with the standard VC (Fig. 2b). This result confirms a previous report [9]. The analysis of the 9 sub-fractions (A − I) from the EtOAc fraction, showed that Fraction E possessed the greatest antioxidant activity (Fig. 2c), while the activity of Fractions A to-C and-, I was negligible (not showed in Fig. 2c). Cataposide, amphicoside and verminoside, were the three major chemical constituents of Fraction E, and all potently scavenged DPPH free radicals. The activity of verminoside was similar toVc (Fig. 2d), while the scavenging activity of veronicoside was weaker than that of the other compounds.

**Fig. 3:**
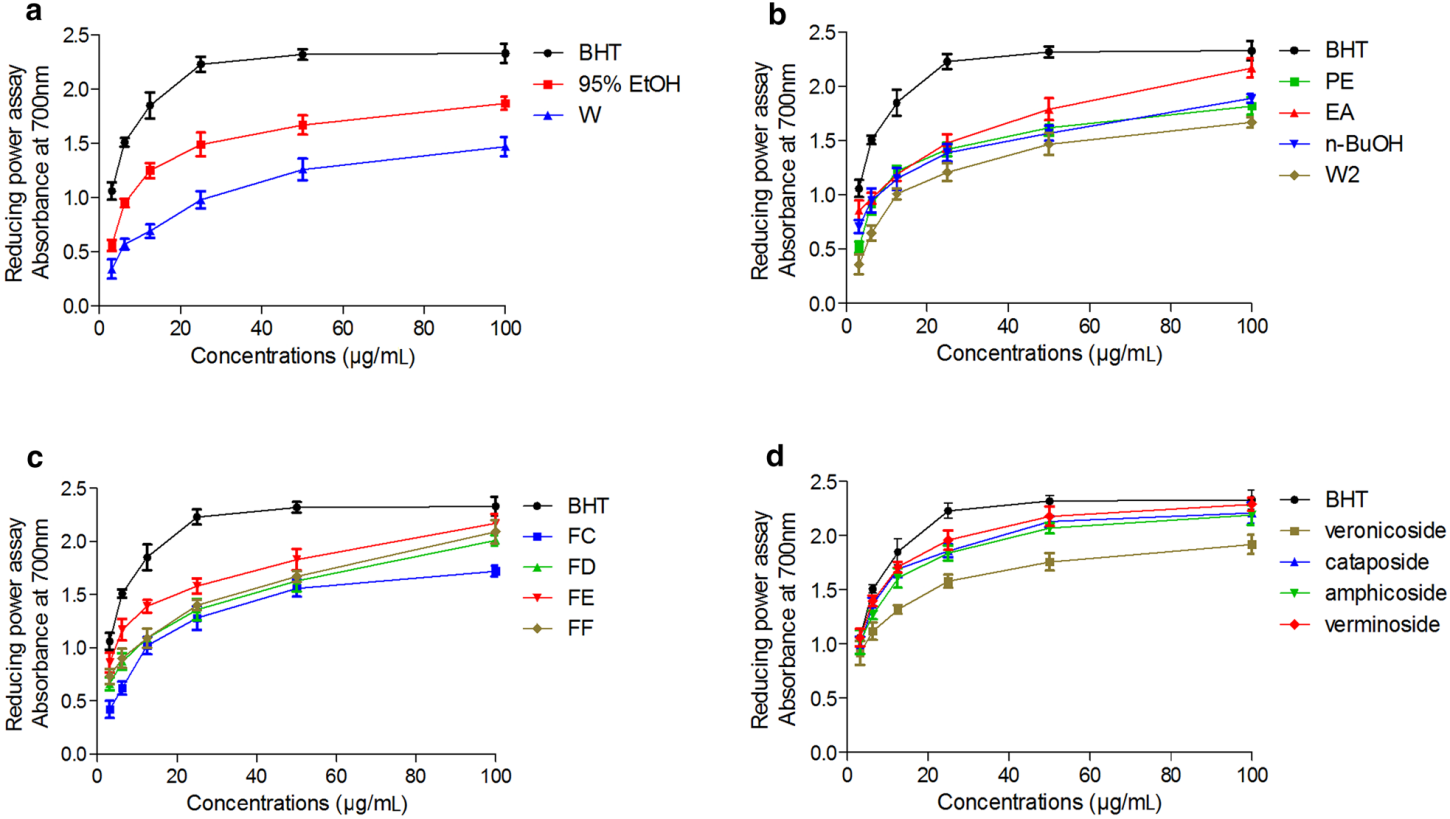
Ferric reducing/antioxidant activity of the **a** 95 % ethanol extract (95 % EtOH), water extract (W); **b** petroleum ether fraction (PE), ethyl acetate fraction (EA), *n*-butanol (*n*-BuOH) fraction, and water fraction (W2) from 95 % ethanol extract; **c** fractions (FD, FE, FF, FG) from EtOAc fraction; and **d** veronicoside, cataposide, amphicoside and verminoside. Results are mean ± SD

The results of the FRAP analysis are shown in Fig. 3. The 95 % ethanol extract had the highest reducing activity, followed by the water extract (Fig. 3a). Among the four fractions, the highest reducing activity was observed in the EtOAc fraction and the lowest in the water fraction (Fig. 3b). Among the 9 sub-fractions (A − I) from the EtOAc fraction, fraction E possessed the highest antioxidant activity (Fig. 3c), while the activity of fraction A, B, H, I was negligible (not showed in Fig. 2c). Cataposide, amphicoside and verminoside, all showed reducing activity and the activity of cataposide similar to Vc (Fig. 3d). However, veronicoside showed slightly lower reducing activity.

As mentioned above, the antioxidant activity of the ethanol extracts was higher than the water extract, and the antioxidant activity order of the four fractions was:water fraction <petroleum ether fraction <n-BuOH fraction <EtOAc fraction. It has been reported that free hydroxyl groups in phenoliccompounds are mainly responsible for antioxidant activity [17]. This may also be the cause of the higher antioxidant activity of cataposide, amphicoside and verminoside which all contain multiple phenolic hydroxylgroups. Additionally, fraction E showed stronger antioxidant activity than cataposide, amphicoside and verminoside at the same concentrations. It is possible that the antioxidant activity of fraction E did not come from any one of these compounds and that it emerged from the interaction of all of these compounds simultaneously.

### In vivo anti-hepatocarcinoma activity assays

**Fig. 4:**
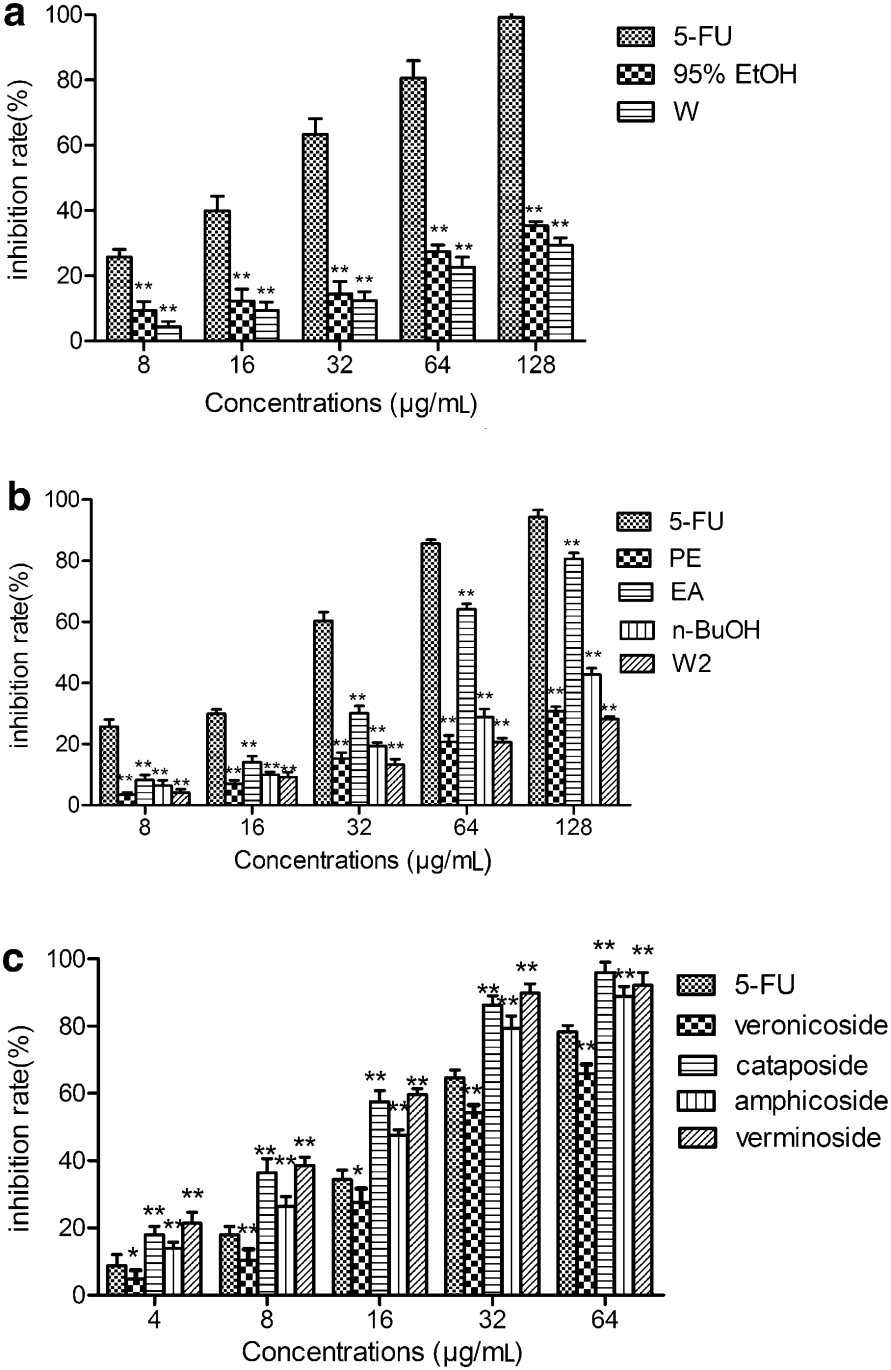
Cell proliferation inhibition rate of **a** 95 % ethanol extract (95 % EtOH), water extract (W); **b** petroleum ether fraction (PE), ethyl acetate fraction (EA), n-butanol (*n*-BuOH) fraction, and water fraction (W2) from 95 % ethanol extract; and **c** veronicoside, cataposide, amphicoside and verminoside. *p < 0.05, **p < 0.01, statistically significant in comparison with control

The ability of each sample to inhibit the cell proliferation of HepG2 cells is shown in Fig. 4. The 95 % ethanol extract possessed a higher cell proliferation inhibition rate than water extract (Fig. 4a).

Although the ethyl acetate fraction showed a lower inhibition rate than 5-fluorouracil, it was higher than the 95 % ethanol extract (Fig. 4b). This result indicated that-, after the 95 % ethanol extract was partitioned into the four fractions, the active compounds were concentrated into the ethyl acetate fraction.

Veronicoside, cataposide, amphicoside and verminoside all strongly inhibited the proliferation of HepG2 cells (Fig. 4c), and the inhibition rate increased in a concentration-dependent manner. The inhibition rates of the compounds, except veronicoside, were much higher than that of 5-fluorouracil. Cataposide and verminoside had a similar suppressive effect on HepG2 cell proliferation and the IC_50_ values of veronicoside, cataposide, amphicoside, verminoside and 5-fluorouracil were 41.25 ± 0.17, 15.54 ± 0.53, 28.32 ± 0.22, 17.82 ± 0.42 and 29.62 ± 0.32 μg/mL, respectively.

The capability of the compounds to hinder proliferation of a cancerous cell line was ascertained by measuring their cytotoxicity in a hepatocarcinoma cell line. The majority of the iridoid glycosides and their derivatives have been described as having an attached aromatic ring. Aromatic rings are cited to be one of the most ‘preferred structures’ to be associated with bioactivities [18]. Hence, as shown in Fig. 4, all the compounds possessed cytotoxic activity. Additionally, cataposide, amphicoside and verminoside inhibited cell proliferation more effectively than veronicoside.We suspected that this was because cataposide, amphicoside and verminoside have more phenolic hydroxyl groups than veronicoside, although their chemical structures are very similar.As reported previously,Picroliv is a standardized mixture obtained from *P. kurroa* and contains at least 60 % of iridoid glycosides. In a number of tests aimed at delineating the anti-hepatotoxic effects of picroliv, it has been shown to have similar or better activity than silymarin [19] 0.5-fluorouracil is a broad-spectrum anti-cancer drug and our data shows that the anti-hepatocarcinoma activities of cataposide, amphicoside, and verminoside were stronger than 5-fluorouracil. Moreover, our work showed that *V. ciliata* Fisch. contains a high amount of iridoid glycosides, indicating that it is potentially valuable as an anti-hepatotoxic drug.

The antioxidant and anti-hepatocarcinoma activities of the ethanol extracts were stronger than those of the aqueous extract, and the ethyl acetate fraction of the 95 % ethanol extract showed the highest activities. Four iridoid glycosides (veronicoside, cataposide, amphicoside, and verminoside) were isolated from the ethyl acetate fraction. All of the compounds exhibited strong antioxidant activity and inhibitory activity on HepG2 cell proliferation. The antioxidant activity of verminoside was equal to Vc. Cataposide, amphicoside and verminoside had stronger anti-hepatocarcinoma activity than 5-fluorouracil.

## Methods

### General

Samples were dissolved in methanol, and electrospray ionization ion trap multiple mass spectrometry (ESI–MS) was performed on a MicrOTOF-Q II (Bruker Daltonics, Germany) plus LC/MS system. UV spectra were obtained using a Perkin-Elmer Lambda 35 spectrometer. ^1^H NMR spectra,^13^C NMR spectra,and 2D NMR (HMBC) spectra were recorded on a Bruker Ascend-400 spectrometer, operating at 400 and 100 MHz for ^1^H and ^13^C, respectively, using MeOD-*d*_4_ as solvents. Chemical shifts were reported in *δ* (ppm) downfield from tetramethylsilane (TMS) as an internal reference, and coupling constants were reported in Hz. Column chromatography (CC) was performed using silica gel (200–300 mesh, 2.4 kg) and Sephadex LH-20. The spots on TLC plates were detected under UV light or by holding under iode vapor, and were visualized by spraying with ethanol-H_2_SO_4_ after heating. Separations by HPLC (LC-3000) were carried out using an Welchrom-C18 column (10 × 250 mm, 5 μm).Unless specified otherwise,the flow rate was 2.0 mL/min.

### Chemicals

5-FU(purity >99 %) was purchased from Chengdu Hua Xia chemical reagent co., LTD, vitaminc(Vc)(purity >99.7 %), 2,6-ditert-butyl-4-methylphenol(BHT)(purity >99.9 %) and Penicillin sodium were purchased from Sigma-Aldrich. Acetonitrile was obtained from Merck. The solvents used for HPLC (high performance liquid chromatography) were of HPLC grade. All other chemicals and reagents used in this study were of analytical grade.

### Plant materials

The herbs of *V. ciliata* Fisch. were purchased from Tibet Tibetan Medicine Group Co., Ltd., China. A voucher specimen (No. 00721478) was identified by Dr. Jie Bai, School of Life Sciences, Sichuan University, and deposited in the Herbarium of Sichuan University.

### Extraction and isolation

**Fig. 5:**
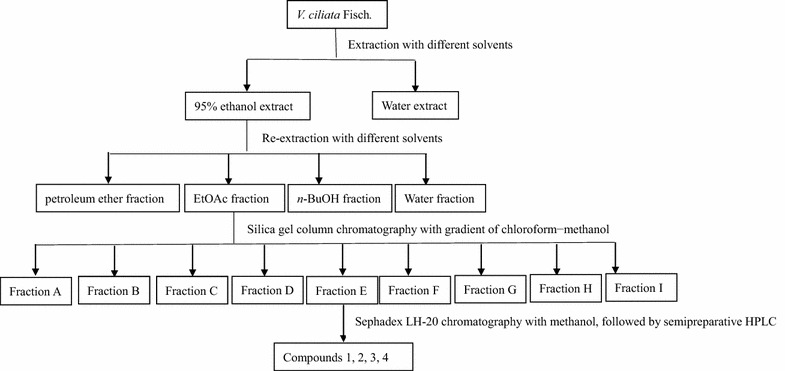
The extraction and isolation procedure of *V. ciliata* Fisch.

The locally collected herbs were shade dried and powdered. The powder (2 kg) was extracted three times at ambient temperature (22–25 °C) with 95 % ethanol. During the extraction with solvents, the solvent was changed every 24 h. The ethanol from the pooled extracts was removed by distillation under reduced pressure at 40–45 °C to create crude extracts (342.5 g). Moreover, the powder (1 kg) was macerated with distilled water (5:1) at room temperature for 12 h, and then boiled for 1 h. After filtration, the extract was dried into a powder by a vacuum-drier at 60 °C to create the extracts (32.6 g). In the biological activity screening tests, the 95 % ethanol extract showed better activity than the water extracts. Therefore, the 95 % ethanol extract was chosen for the following isolation. The extracted solutions (342.5 g) were suspended in distilled water, and then sequentially extracted three times (1500 mL × 3) with petroleum ether, ethyl acetate (EtOAc) and *n*-butanol (n-BuOH) to produce petroleum ether-soluble (11.2 g), EtOAc (127.3 g), *n*-BuOH (24.9 g) and water (69.5 kg) extracts, respectively. Among these fractions, the EtOAc-soluble fraction was found to have the highest antioxidant and anti-hepatocarcinoma activity. Therefore, the EtOAc-soluble fraction (100 g) was submitted on a silica gel column (10 × 100 cm) using a gradient of chloroform–methanol (6L) 100:0, 98:2, 96:4, 94:6, 92:8, 90:10, 80:20, and 70:30. Fractions of 6L were collected and combined after TLC analysis to yield 9 fractions (A – I). Fraction E (18.08 g)possessed the highest antioxidant and anti-hepatocarcinoma activity and was consecutively re-chromatographed on SephadexLH-20 (5 × 70 cm, 760 g) using a gradient of methanol-H2O (2700 mL) 100:0,80:20,60:40,40:60,20:80. Fractions of 100 mL were collected and combined after TLC analysis to yield 4 fractions [fraction1 (0.45 g), fraction 2 (6 g), fraction 3 (2.1 g) and fraction 4 (3 g)]. These fractions were followed by semi-preparative HPLC using 55 % methanol solution as the mobile phase to obtain compounds 1 (22 mg), 2(67 mg), 3 (27 mg), and 4 (31 mg).The extraction and isolation procedure of *V. ciliata* Fisch. is shown in Fig. 5.

The spectroscopic data were listed below

Veronicoside (compound 1) was obtained as a white amorphous powder. ESI–MS (positive) m/z: 489[M + Na]^+^; ESI–MS (negative)m/z: 465[M-H]^−^; ^1^H-NMR (400 MHz, CH_3_OH-*d*_*4*_) *δ*: 2.48(1H, dd, *J* = 9.0, 7.0 Hz, H-9), 2.59 (1H, m, H-5), 3.0 ~ 3.24 (4H, m, H-2′,3′,4′,5′), 3.46 (1H, m, H-6′b), 3.74 (1H, brs, H-7), 3.77 (1H, m, H-6′a), 3.77 (1H, m, H-10b), 3.95 (1H, dd, *J* = 13.3, 4.8 Hz, H-10a), 4.65 (1H, d, *J* = 8.0 Hz, H-1′), 5.03 (1H, m, H-4), 5.14 (1H, m, H-6), 5.14 (1H, d, *J* = 9.5 Hz, H-1), 6.45 (1H, d, *J* = 6.5 Hz, H-3), 7.58 (2H, t, *J* = 8.0 Hz, H-3′′, 5′′), 7.72 (1H, t, *J* = 7.5 Hz, H-4′′), 8.04 (2H, d, *J* = 8.5 Hz, H-2′′, 6′′); ^13^C-NMR (100 MHz, CH_3_OH-*d*_*4*_): see Table 1.

Cataposide (compound 2) was obtained as a white amorphous powder. ESI–MS (positive) m/z: ESI–MS m/z: 483 [M + H]^+^; ^1^H-NMR (400 MHz, CH_3_OH-*d*_*4*_) *δ*: 2.49 (1H, m, H-9), 2.57 (1H, m, H-5), 3.0-3.23 (4H, m, H-2′, 3′, 4′, 5′), 3.42 (1H, dd, *J* = 11.8, 6.8 Hz, H-6′b), 3.68 (1H, d, *J* = 1.5 Hz, H-7), 3.71 (1H, dd, *J* = 11.8, 1.8 Hz, H-6′a), 3.72 (1H, d, *J* = 13.0 Hz, H-10b), 3.92 (1H, d, *J* = 13.5 Hz, H-10a), 4.63 (1H, d, *J* = 8.0 Hz, H-1′), 4.97 (1H, dd, *J* = 6.0, 4.5 Hz, H-4), 5.07 (1H, dd, *J* = 8.0, 1.0 Hz, H-6), 5.12 (1H, d, *J* = 9.5 Hz, H-1), 6.43 (1H, dd, *J* = 5.5, 1.5 Hz, H-3), 6.86 (2H, d, *J* = 9.0 Hz, H-3′′, 5′′), 7.86 (2H, d, *J* = 8.5 Hz, H-2′′, 6′′); ^13^C-NMR (100 MHz, CH_3_OH-*d*_*4*_): see Table 1.

Amphicoside (compound 3) was obtained as a white amorphous powder. ESI–MS (positive) m/z: 535 [M + Na]^+^; ESI–MS (negative)m/z: 511[M-H]^−^; ^1^H-NMR (400 MHz, CH_3_OH-*d*_*4*_) *δ*: 2.63 (1H, m, H-9), 2.68 (1H, m, H-5), 3.23–3.43 (4H, m, H-2′,3′, 4′, 5′), 3.65 (1H, dd, *J* = 12.0, 6.5 Hz, H-6′b), 3.75 (1H, d, *J* = 1.0 Hz, H-7), 3.85 (1H, d, *J* = 13.0 Hz, H-10b), 3.90 (3H, s, OCH_3_), 3.93 (1H, dd, *J* = 12.0, 2.0 Hz, H-6′a), 4.21 (1H, d, *J* = 13.5 Hz, H-10a), 4.80 (1H, d, *J* = 8.0 Hz, H-1′), 5.01 (1H, dd, *J* = 5.8, 4.3 Hz, H-4), 5.11 (1H, dd, *J* = 8.3, 1.3 Hz, H-6), 5.20 (1H, d, *J* = 9.5 Hz, H-1), 6.38 (1H, dd, *J* = 6.0, 1.5 Hz, H-3), 6.87 (1H, d, *J* = 8.5 Hz, H-5′′), 7.57 (1H, d, *J* = 2.0 Hz, H-2′′), 7.60 (1H, dd, *J* = 8.5, 2.0 Hz, H-6′′); ^13^C-NMR (100 MHz, CH_3_OH-*d*_*4*_): see Table 1.

Verminoside (compound 4) was obtained as a white amorphous powder. ESI–MS (positive) m/z: 547[M + Na]^+^; ESI–MS (negative)m/z: 523[M-H]^−^; 1H-NMR (400 MHz, CH_3_OH-*d*_*4*_) *δ*: 2.61 (1H, m, H-5), 2.63 (1H, m, H-9), 3.26 ~ 3.45 (4H, m, H-2′,3′, 4′, 5′), 3.66 (1H, dd, *J* = 12.0, 6.5 Hz, H-6′b), 3.70 (1H, brd, *J* = 1.0 Hz, H-7), 3.83 (1H, d, *J* = 13.0 Hz, H-10b), 3.92 (1H, dd, *J* = 11.8, 1.8 Hz, H-6′a), 4.16 (1H, d, *J* = 13.0 Hz, H-10a), 4.80 (1H, d, *J* = 8.0 Hz, H-1′), 5.1 (1H, dd, *J* = 6.0, 4.0 Hz, H-4), 5.02 (1H, dd, *J* = 7.8, 1.3 Hz, H-6), 5.16 (1H, d, *J* = 9.0 Hz, H-1), 6.31 (1H, d, *J* = 15.5 Hz, H-8′′), 6.38 (1H, dd, *J* = 6.0, 1.5 Hz, H-3), 6.81 (1H, d, *J* = 8.0 Hz, H-5′′), 6.98 (1H, dd, *J* = 8.5, 2.0 Hz, H-6′′), 7.07 (1H, d, *J* = 2.0 Hz, H-2′′), 7.61 (1H, d, *J* = 16.0 Hz, H-7′′); ^13^C-NMR (400 MHz, CH_3_OH-*d*_*4*_): see Table 1.

### Assays for antioxidant activity

The antioxidant activities of the 95 % ethanol extract, and water extract of *V. ciliata* Fisch. were measured. Next, the 95 % ethanol extract was further divided into petroleum ether, ethyl acetate, *n*-butanol, and water fractions, and the antioxidant activities of each fraction were compared. The activities of 9 fractions and 4 pure compounds isolated from the ethyl acetate fraction were also determined.

### DPPH radical scavenging assay

The scavenging activity of the DPPH radical was evaluated according to an improved DPPH assay [20] with slight modifications. Briefly, 2 mL of the samples at different concentrations (3.25–100 μg/mL, dissolved in ethanol) were mixed with 2 mL of DPPH solution (0.1 mM, in ethanol). VC was used as a comparison. Then, the mixtures were shaken evenly and allowed to stand at room temperature in the dark for 30 min before the absorbance was measured at 517 nm. The radical scavenging activity was calculated as follows: DPPH radical scavenging activity (%) = [1 − (Ai − As)/Ac] ×100, where Ac is the absorption of the negative control, Ai represents the absorption of the experiment group and As represents the absorption of the sample background. The concentration of samples reducing 50 % of free radical DPPH (IC_50_) was determined by plotting the percentage of inhibition against the sample concentrations.

### Reducing power assay

The reducing power of the samples was measured using a previous method [21]. Briefly, 1.0 mL of samples solutions at different concentrations(3.25–100 μg/mL, dissolved in ethanol) was mixed with 2.5 mL of phosphate buffer saline (0.2 M, pH 6.6) and 2.5 mL of 1 % (w/v) K_3_Fe (CN)_6_ solution. After incubation at 50 °C for 30 min, 2 mL of 10 % trichloroacetic acid (TCA) was added. Then 2.0 mL of the upper layer was combined with 2.0 mL of distilled water and 1 mL of 0.1 % (w/v) FeCl_3_ solution. The absorbance was analyzed at 700 nm (BHT was used as a positive control). Increased absorbance of the reaction mixture indicates a greater reducing power.

### Anti-hepatocarcinoma activity

#### Cell culture

Human hepatocellular carcinoma HepG2 cells were obtained from the cell bank of the Chinese Academy of Sciences. The cells were cultured in RPMI 1640 medium (Gibco BRL) supplemented with 100 IU/mL penicillin, 100 IU/mL streptomycin, and 0.01 mg/mL fetal bovine serum (FBS) and were incubated at 37 °C in a humidified incubator with an atmosphere of 5 % CO_2_.

#### Cell proliferation inhibition assay

The effect of each sample on the proliferation of HepG2 cells was estimated using the 3-(4, 5-dimethylthiazol-z-yl)-2, 5-diphenyl tetrazolium bromide (MTT) test [22] which is based on premise that succinate dehydrogenase in the mitochondria of living cells can cleave the tetrazolium ring of MTT to produce formazan. HepG2 cells in the exponential growth phase, at a density of 5 × 10^3^ cells/mL, were seeded in 96-well culture plates (100 μL/well) and incubated overnight. 20 h after incubation, the cells were treated with various concentrations of samples. After 72 h, the cells were washed with fresh medium, treated with MTT solution and incubated for an additional 3 h. The formazan crystals were dissolved in 100 μL of SDS solution, and the optical density (OD) was measured at 570 nm using a microplate reader. The results are based on at least three independent experiments performed in quadruplicate. The positive control was 5-fluorouracil, and cells that were not treated with a sample were used as a control. Cell proliferation inhibition rate (CPIR) was identified and calculated using the following formula:

$${\rm{Cell\,proliferation\,inhibition\,rate}}\,= \,\left[ {{\rm{1}}\,-\,{\rm{O}}{{\rm{D}}_{{\rm{sample}}}}/{\rm{O}}{{\rm{D}}_{{\rm{control}}}}} \right]\,\times\,{{1}}00$$

### Statistical analysis

All of the results were expressed as mean ± standard deviation (SD). Statistical differences of experimental data among groups were tested using one-way ANOVA (n = 3) analysis or paired two-sample *t* test (n = 3) analysis (SPSS 15.0, SPSS Inc., Chicago, IL, USA). Statistical significance was set at *p* < 0.05.

## Conclusions

Four iridoid glycosides, (veronicoside, cataposide, amphicoside and verminoside) were isolated from the extract of *V. ciliata* Fisch. using bioassay-guided screening. Among these compounds, veronicoside and verminoside were isolated for the first time from this plant. The above results indicated that these compounds were the active chemical components responsible for the antioxidant and anti-hepatocarcinoma properties of *V. ciliata* Fisch. The underlying mechanism of their bioactivity is worthy of further investigation.
